# Biomechanical evaluation of a fixation technique with a modified hemicerclage for tibial tuberosity transposition: an *ex vivo* cadaveric study

**DOI:** 10.3389/fvets.2024.1375380

**Published:** 2024-05-09

**Authors:** Pavlos Natsios, Rahel Capaul, Norbert Kopf, Antonio Pozzi, Selena Tinga, Brian Park

**Affiliations:** ^1^Clinic for Small Animal Surgery, Vetsuisse Faculty, University of Zurich, Zurich, Switzerland; ^2^Tierklinik Breitensee, Vienna, Austria; ^3^Department of Clinical Sciences, College of Veterinary Medicine, Cornell University, Ithaca, NY, United States

**Keywords:** TTT, MPL, dog, patellar luxation, tibial tuberosity fixation

## Abstract

**Objectives:**

(1) To determine stiffness, load at failure, and mode of failure of a novel fixation method with a tension modified-hemicerclage (MH) for tibial tuberosity transposition, and (2) to compare the biomechanical properties of this novel fixation technique to 2 pins (2Pins) and 2 pins and tension band wire fixation (2Pins + TBW).

**Study design:**

Thirty cadaveric stifles from dogs between 5.2 and 13.1 kg were assigned to 3 treatment groups: Group 1: fixation technique with MH; Group 2: 2Pins; Group 3: 2Pins + TBW. Biomechanical testing was performed with the tibia positioned at an angle of 135° relative to vertical position. Tensile force was applied to the patellar ligament until catastrophic failure was observed. The mode of failure, the load at failure, and the stiffness were compared among treatment groups.

**Results:**

The mean stiffness of the novel fixation (38.1 N/mm ± 7.1) and the 2Pins + TBW (40.2 N/mm ± 9.3) were greater than the 2Pins (26.7 N/mm ± 6.7). There was no significant difference between the novel fixation technique and 2Pins + TBW in stiffness and maximum load to failure. The 2Pins (284.3 N ± 70.5) failed at a significantly lower load than the tension modified-hemicerclage (555.7 N ± 225.9 N) and 2Pins + TBW (715.3 N ± 339.8 N).

**Conclusion:**

A fixation technique using a modified hemicerclage had the same strength and stiffness as the 2Pins + TBW and was stronger and stiffer than the 2 Pins technique in a cadaveric model.

## Introduction

Patellar luxation is one of the most common hindlimb orthopedic diseases in dogs (1–5). Patellar luxation can be medial, lateral, or bidirectional, with the vast majority being medial (1, 2, 6, 7). Small breed dogs are 12 times more likely to suffer from the disease and are affected bilaterally in 52 to 65% of cases (7). The pathogenesis of congenital patellar luxation is still unclear, but malalignment of the quadriceps mechanism is considered a major predisposing factor (8–10).

Surgical treatment is recommended for the symptomatic patient (6, 7). Tibial tuberosity transposition (TTT) is performed to realign the quadriceps mechanism (6, 9, 11–13) and reduce the risk of reluxation (2, 14, 15). Several techniques have been suggested for the fixation of the TTT, including 2 pins and tension band wire (2Pins + TBW) or 2 pins (2Pins). Previous studies suggested that the pins should be placed approximately perpendicular to the long axis of the tibia in order to best counteract the tensile force of the patellar tendon (15–19).

The reported implant-associated complication rate of TTT for MPL varies from 18 to 43%, with a high rate of major complications (14, 15, 17, 20–23). Specifically, complications can include implant breakage, implant migration, and avulsion of the tibial tuberosity, as well as infection (15, 24). Most of the complications are associated with the smooth pins placed from cranial to caudal to stabilize the tuberosity fragment to the main tibial segment, which can cause soft tissue irritation or can migrate (14, 15, 17, 20, 23, 25, 26). A fixation technique of the tibial tuberosity that does not require pins might be beneficial to reduce the rate of complications of TTT.

A TTT technique with a single-length orthopedic wire has been proposed to avoid complications associated with the use of pins (27). The proposed technique utilizes a single-length orthopedic wire placed as a modified hemicerclage (MH) in a tension band fashion, without the use of pins through the tuberosity. Similar studies employing quasi-static load-to-failure tests have been conducted, focusing on different fixation methods, however predominantly involving larger breed subjects (12, 18, 28–30). This biomechanical study explicitly focuses on the initial validation of the novel technique’s biomechanical properties compared to well-established techniques and aims to primarily assess the mechanical strength of the fixation method.

The objectives of the study are (1) to determine the stiffness, load to failure, and mode of failure of a novel fixation method for TTT with a single-length orthopedic wire placed as a modified hemicerclage in tension band fashion (MH) in a small dog model, and (2) to compare these biomechanical testing results of the novel fixation to other tibial tuberosity fixation techniques (2Pins and 2Pins + TBW). We hypothesized that (1) there would be no difference in stiffness and load to failure between the novel fixation technique and the 2Pins + TBW and that (2) the novel fixation technique would fail at a higher load and be stiffer compared to the 2Pins fixation method.

## Materials and methods

Thirty tibias (*n* = 30) from skeletally mature small-breed dogs, euthanatized for reasons unrelated to this study, were collected for the study. All the cadaveric specimens were donated for research purposes by their owners, and written consent was obtained according to the institute and national regulations. Tibias from chondrodystrophic dogs or dogs with any stifle pathology were excluded from the study. Specimens underwent radiographic evaluation prior to testing to confirm skeletal maturity and to exclude any stifle pathologies. Immediately after euthanasia, all tibial specimens were dissected free from the soft tissues except the patellar tendon leaving the patella attached and wrapped in saline-soaked towels, then stored at −20° C for subsequent use. Prior to testing, the tibial specimens were thawed at room temperature for 24 h.

### Surgical technique

Each tibia was randomly assigned to 1 of 3 groups representing three different TTT fixation methods. In Group 1 (*n* = 10), the TTT was fixed using the novel fixation technique using a single orthopedic wire used as a modified hemicerclage in a tension band fashion (0.6 mm, 316LVM stainless steel) (DePuy Synthes Vet, Oberdorf, Switzerland). In Group 2 (*n* = 10), the TTT was fixed using 2 pins (1.0 mm, 316LVM stainless steel) (DePuy Synthes Vet, Oberdorf, Switzerland) that were horizontally aligned. In Group 3 (*n* = 10), the TTT was fixed using 2 pins (1.0 mm, 316LVM stainless steel) (DePuy Synthes Vet, Oberdorf, Switzerland) that were horizontally aligned to each other with an added TBW (0.6 mm, 316LVM stainless steel) (DePuy Synthes Vet, Oberdorf, Switzerland).

**Figure 1:**
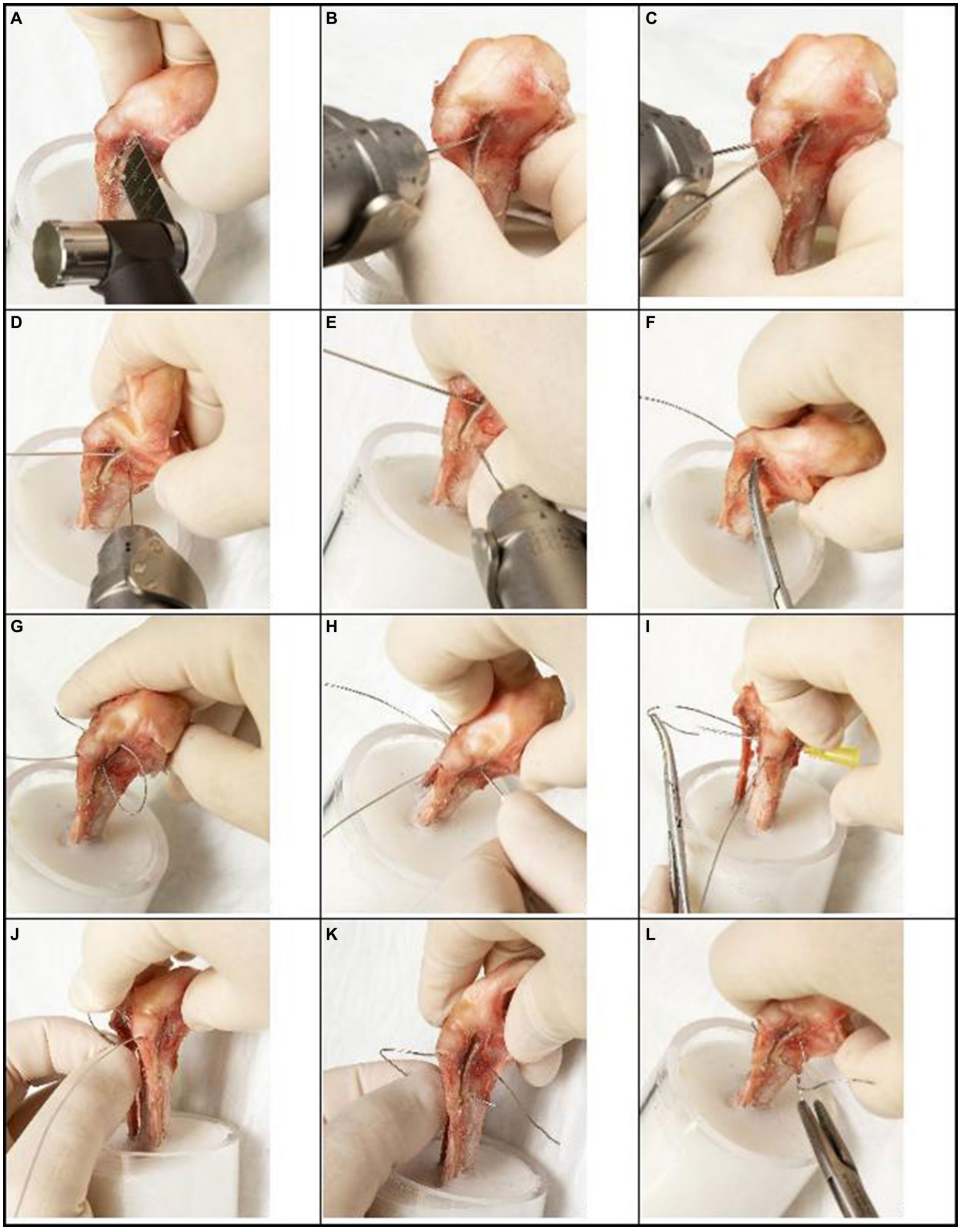
Step-by-step guide for novel TTT fixation technique (tension modified-hemicerclage) applied to a canine right tibia. A clinician may modify this technique in direction and magnitude of translation as needed for treatment of individual cases. **(A)** An osteotomy of the tibial tuberosity is made, extending from cranial of the intermeniscal ligament, through a point 5 mm caudal to the insertion of the patellar ligament and extending to the distal crest, leaving the periosteal bridge intact. **(B)** The osteotomized segment is translated laterally by 1 mm, and a 1.0 mm K-wire is placed medial to the tuberosity as temporary stabilization until final fixation is performed. **(C)** The first hole is drilled with a 1.0 mm K-wire in a craniocaudal direction, through the tibial tuberosity at the insertion of the patellar ligament. **(D)** The second hole is drilled with a 1.0 mm K-wire in a mediolateral direction (bicortically), 2 mm caudal to the osteotomy at the level of the insertion of the patellar ligament. **(E)** The third hole is drilled with a 1.0 mm K-wire in a mediolateral direction (bicortically), 2 mm caudal to the distal part of the tibial crest. **(F,G)** A 0.6 mm wire is passed through the first hole and retrieved through the osteotomy initially on the medial side and then passed on the lateral side of the tibia. **(H–J)** The wire is then passed through the second hole from lateral to medial with the help of a 20 Gage needle. **(K)** The end of the wire extending from the cranial aspect of the osteotomized tibial tuberosity is then passed through the third hole from lateral to medial. **(L)** The two ends of the wire are secured on the medial aspect of the tibia with four twists.

All surgeries were performed by a single surgeon (P.N.) with experience in the three fixation techniques. The osteotomies were standardized by defining the proximal and distal landmarks. Proximally, the osteotomy started cranial to the intermeniscal ligament. Distally, the second point marked for the osteotomy was positioned 5 mm caudal to the insertion of the patellar ligament on the tibial tuberosity. The osteotomy was performed with an oscillating saw, connecting the two points and ending at the distal crest, leaving a bridge of periosteum intact (Figure 1A). The osteotomized tuberosity segment was then translated laterally by 1 mm, measured at the level of the insertion of the patellar ligament on the tibia, and fixated according to the specimen’s assigned treatment group (Figure 1B).

#### Group 1: tension modified-hemicerclage technique

**Figure 2:**
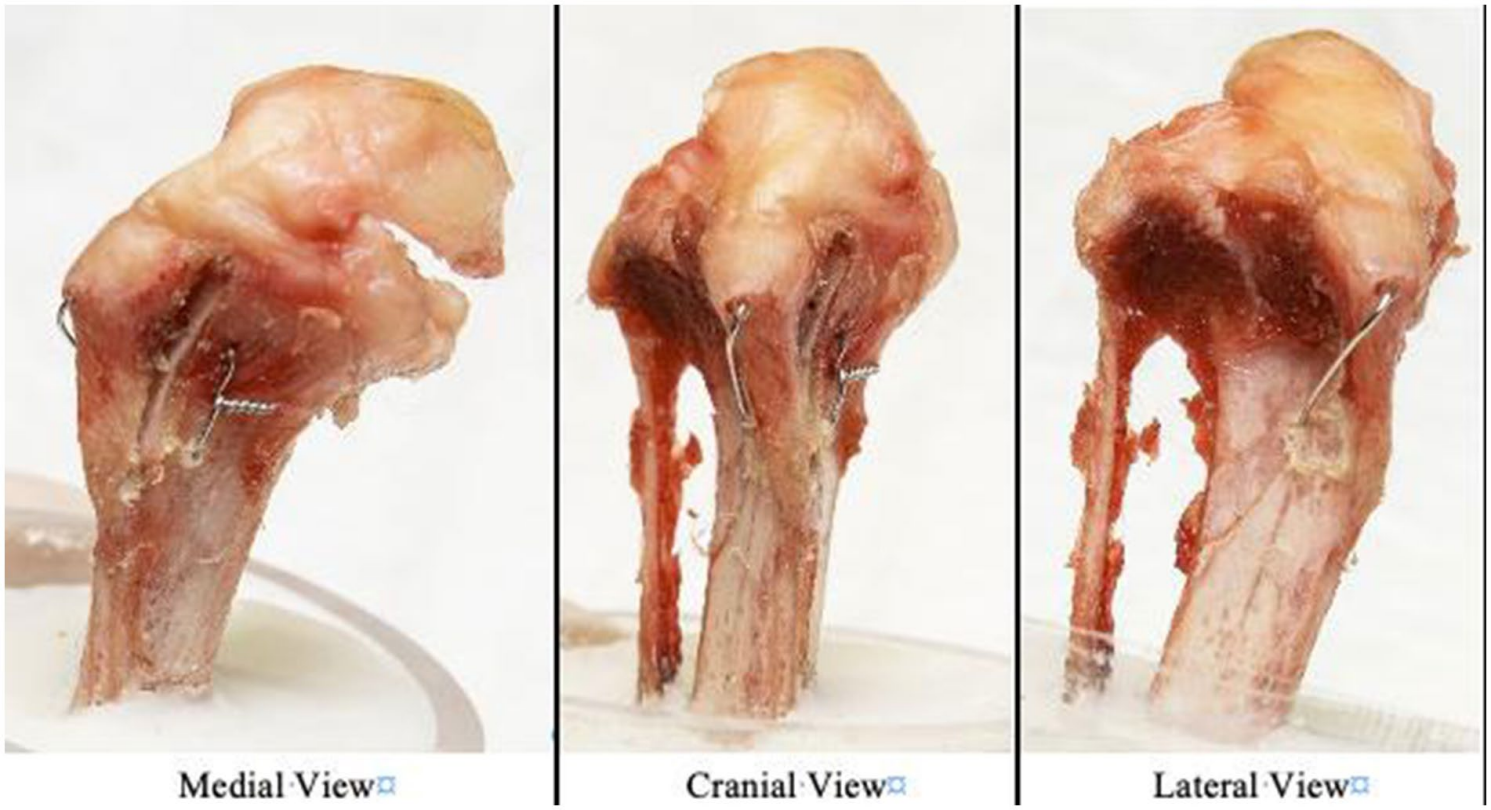
Photographs of the modified hemicerclage in tension band fashion applied to a canine right tibia and observed from three views (medial, cranial, and lateral view).

**Figure 3:**
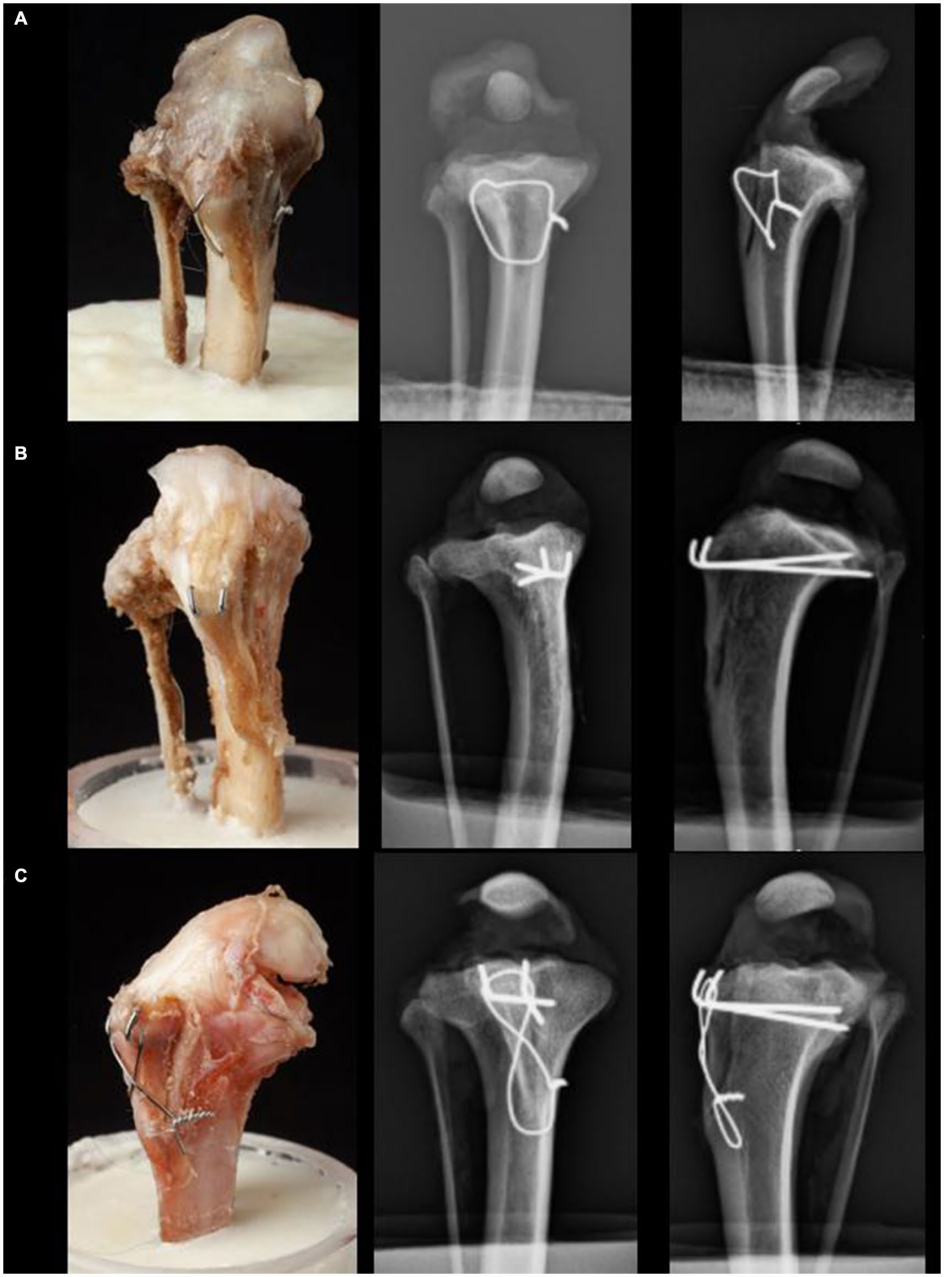
Craniocaudal and lateral radiographs of the three different fixation techniques for TTT: **(A)** Group 1 is fixated with the novel fixation technique using a single orthopedic wire as a modified hemicerclage in tension band fashion. **(B)** Group 2 is fixated with 2 Pins. **(C)** Group 3 is fixated with 2 Pins and a figure-eight TBW.

After the completion of the osteotomy, three holes were predrilled on the tibia with a 1 mm K-wire. The first hole was created at the insertion of the patellar ligament in a craniocaudal direction through just the tuberosity segment (approximately 5 mm depth) (Figure 1C). The second hole was created in the mediolateral direction through both cortices of the main tibial segment, 2 mm caudal to the osteotomy, and at the same proximodistal level as the first hole (Figure 1D). The third hole was also drilled from medial to lateral through both cortices of the main tibial segment, positioned 2 mm caudal to the distal part of the tibial crest (Figure 1E). An orthopedic wire (0.6 mm 316LVM stainless steel) (DePuy Synthes Vet, Oberdorf, Switzerland) was then passed through the first hole and retrieved on the lateral aspect of the tibia and passed through the second hole to the medial side (Figures 1F–J). The wire’s free end was guided from the osteotomized tibial tuberosity. It passed through the third hole, going from the lateral side to the medial side of the leg. On the medial side of the tibia, the wire was secured and tensioned until it became taut, with four remaining twists (Figures 1K,L, 2, 3A).

#### Group 2: two horizontally aligned pins technique (2Pins)

After completion of the osteotomy, the pins (1.0 mm 316LVM stainless steel) (DePuy Synthes Vet, Oberdorf, Switzerland) were placed into the tibial tuberosity at the insertion of Sharpey’s fibers, located at 1/3 and 2/3 the width of the tuberosity in the mediolateral direction, and directed in a caudoproximal direction, exiting the caudomedial cortex of the tibia 5–10 mm distal to the joint. After each pin exited the caudal cortex of the bone, it was retracted cranially by a distance of 5 mm. Subsequently, it was securely grasped at the level of the cranial cortex using a heavy needle holder (Aesculap GmbH, Tuttlingen, Germany), ensuring it lay flush with the bone surface. Utilizing a pin bender (DePuy Synthes Vet, Oberdorf, Switzerland), the pins were then bent in a proximal direction against the resistance provided by the heavy needle holder. This adjustment was followed by a controlled reinsertion of the pins into the bone, resulting in their emergence from the caudal cortex. The protruding ends of the pins were then trimmed, ensuring that a residual length of approximately 2 mm remained, oriented parallel to and in contact with the surface of the bone, lying flat against it (Figure 3B).

#### Group 3: two horizontally aligned pins and tension band wire (2Pins + TBW)

The 2 pins were placed as described for the 2Pins group, then a TBW with four twists was applied, and the pins were bent as described for the 2Pins group. The bone tunnel for the TBW was drilled from medial to lateral through the main tibial segment using a 1 mm K-wire (DePuy Synthes Vet, Oberdorf, Switzerland), 2 mm caudal to the cranial cortex of the tibia. This TBW tunnel was positioned distal to the tibial tuberosity by the same distance as the length of the patellar ligament (18). The TBW was positioned in a figure-eight formation around the cranial aspect of the tibial crest and then tightened. After securing it, the excess wire was cut off, leaving four twists on the medial aspect of the tibia (Figure 3C).

### Biomechanical testing

**Figure 4:**
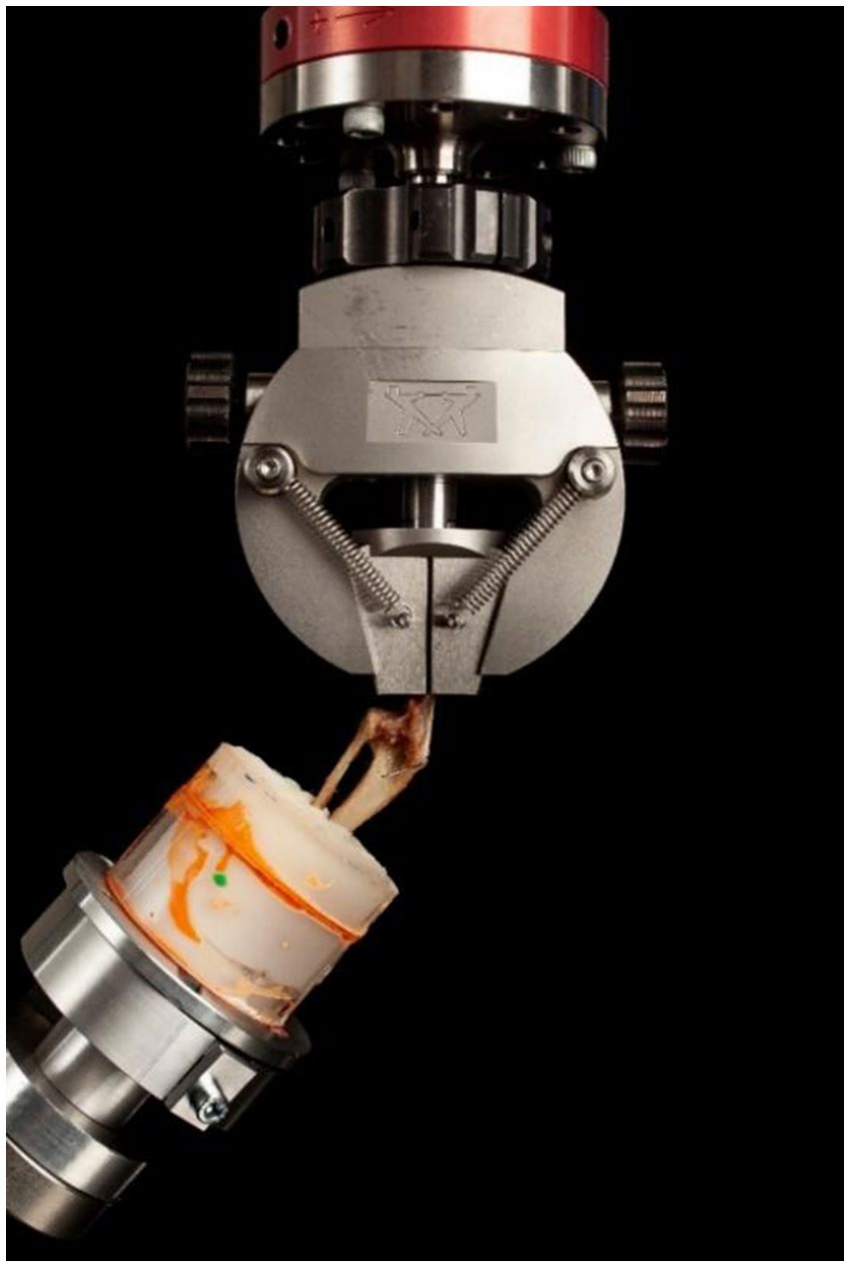
Biomechanical testing setup. The specimens were potted in PMMA and clamped at an angle of 135° to mimic the patellar ligament angle of dogs in the midstance, weight-bearing phase. Following the application of the pre-load force of 10 N, a tensile load was applied at a rate of 5 N per second until failure was reached.

All specimens were potted in cylindric plastic tubes (52 mm diameter, 6 cm height) with PMMA (polymethyl methacrylate), leaving 5 cm of the tibia exposed proximal to the PMMA. The specimen potting utilized an alignment jig, ensuring correct alignment with laser markers to prevent varus/valgus deviations. For mechanical testing, the patellar ligament was tensioned vertically, and the tibia was positioned with a custom-made metallic jig at an angle of 135° relative to vertical position (Figure 4). A vertical tensile force was applied to the patellar ligament at a rate of 5 N/s after a pretension of 10 N (18). Force, and displacement data were recorded, and stiffness was calculated using the formula: *Stiffness =* $\Delta \;\mathrm{Force}\;\left(N\right)/\Delta \;\mathrm{Displacement}\;(\mathrm{mm}$ ). The stiffness was calculated using data from the 30 to 60% range of the curve’s elastic region. Two different points of failure were examined. Video documentation was synchronized with the tensile force documentation in order to verify the link between failure and numerical values. The first point of failure was defined as failure of the periosteal bridge, as indicated by the first drop of the tensile force, verified by synchronized video documentation. The second point of failure was defined as complete failure of the osteotomy, as indicated by a second drop in tensile force, and visualized as a complete detachment of the osteotomy fragment on synchronized video documentation.

### Statistical analysis

All statistical calculations were performed using commercially available statistical software (SPSS IBM Corp. Armonik, NY).^1^ First, the data were evaluated for normal distribution and equal variance using the Shapiro–Wilk test and Levene’s F test. The data were normally distributed with equal variance, so a one-tailed independent two-sample t-test was used to compare stiffness and maximum load at the two failure points. A *p*-value less than 0.05 was considered statistically significant for all analyses.

## Results

All cadaveric specimens in this study were skeletally mature dogs, including nonchondrodystrophic mixed breeds (*n* = 25), Poodle (*n* = 3), and Fox Terrier (*n* = 2), with median weight 7.7 kg (range = 5.2 kg – 13.1 kg) (mean weight = 7.4 kg).

### Stiffness

The mean stiffness values, range, and standard deviation for all groups are reported in Table 1. The 2Pins group was on average 31 and 33% less stiff than the MH group (*p* = 0.0015) and the 2Pins + TBW, respectively (*p* = 0.0016 respectively). There was no difference in stiffness between the MH group and the 2Pins + TBW group (*p* = 0.60) (Table 1).

**Table 1:** Summary of the outcomes of biomechanical testing between the 3 treatment groups [Mean ± standard deviation (range)].

	Group 1 Tension modified-hemicerclage (MH)	Group 2 2Pins	Group 3 2Pins + TBW
Specimen	10	10	10
Stiffness (N/mm)	38.2 ± 7.1 (24.7–48.3 N/mm)	26.7 ± 6.70* (15.3–34.4 N/mm)	40.2 ± 9.3 (28.2–55.8 N/mm)
Periosteal bridge failure load (*N*)	449.2 ± 263.5 (204.5–997.0 N)	138. 1 ± 77.7* (43.0–296.0 N)	488.7 ± 378.7 (131.0–1507.2 N)
Max. load at failure (*N*)	555.7 ± 225.9 (269.0–997.0 N)	284.4 ± 70.5* (201.0–426.2 N)	715.3 ± 339.8 (370.0–1507.2 N)
Main mode of failure	Severing the osteotomy fragment by the cerclage	Pulling out of the pins	Breaking of the TBW and pulling out of the pins

There are statistically significant results in the comparisons between Groups 1&3 and Group 2, which are denoted by (*).

### Load to failure of periosteal bridge

**Figure 5:**
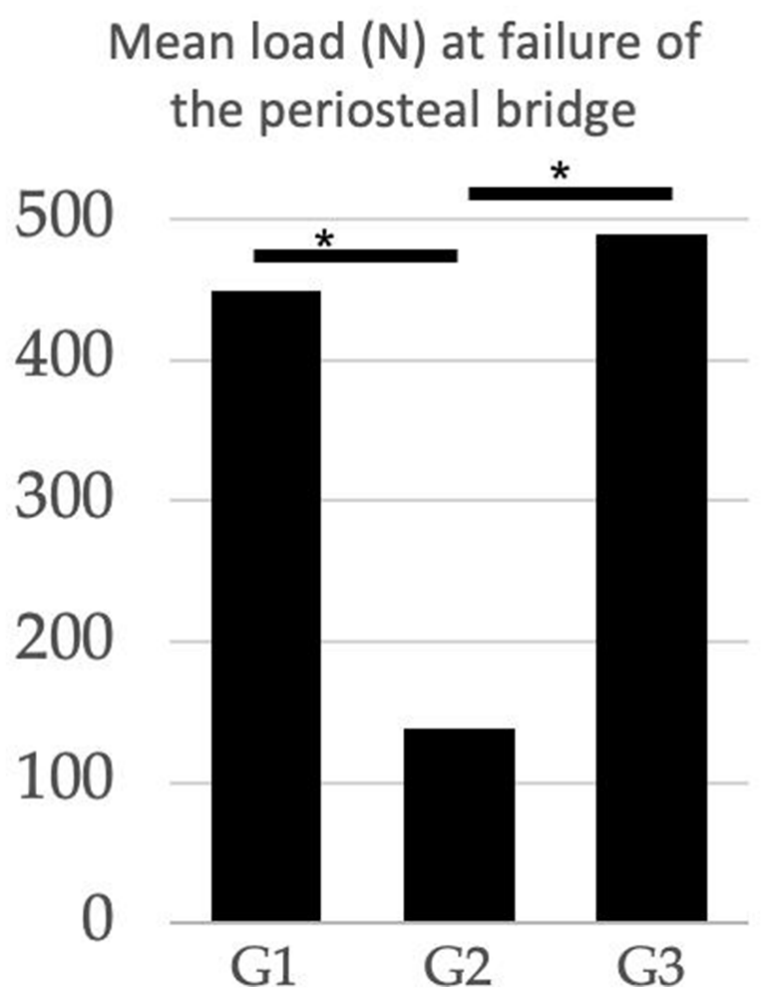
Comparison of the mean load (*N*) at failure of the periosteal bridge between treatment groups. G1 (modified hemicerclage in tension band fashion) = 449.2 N; G2 (2Pins) = 138.1 N; G3 (2Pins + TBW) = 488.7 N. The 2Pins group experienced periosteal failure at lower load than the other 2 treatment groups. (*Statistically significant results).

The mean load to failure values, range, and standard deviation for all groups are reported in Table 1. The load to the first failure point (failure of the periosteal bridge) for 2Pins was 69% (*p* = 0.001) and 78% (*p* = 0.005) lower than the MH group and 2Pins + TBW, respectively. There was no difference in load between the MH and 2Pins + TBW (*p* = 0.39) (Figure 5).

### Maximum load to failure

**Figure 6:**
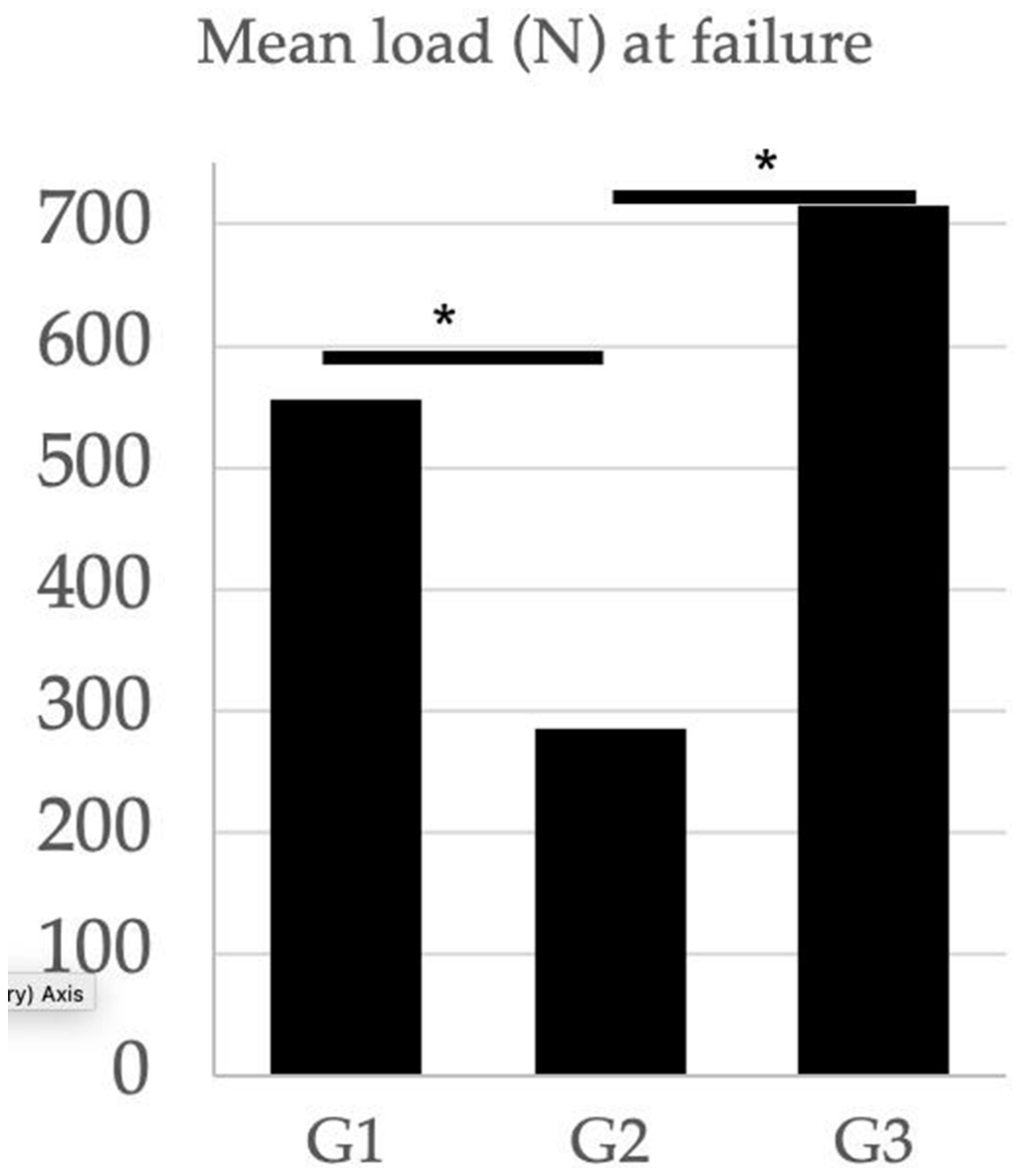
Comparison of the mean load (*N*) at complete failure between treatment groups. G1 (modified hemicerclage in tension band fashion) = 555.7 N; G2 (2Pins) = 284.4 N; G3 (2Pins + TBW) = 715.3 N. The 2 pin group experienced complete failure at lower load than the other 2 treatment groups. (*Statistically significant results).

The mean maximum load to failure values, range, and standard deviation for all groups are reported in Table 1. The maximum load to failure of the construct for 2Pins was 49% (*p* = 0.001) and 60% (*p* = 0.0005) lower than the MH and 2Pins + TBW, respectively. No difference was observed between the MH and 2Pins + TBW group (*p* = 0.12) (Figure 6).

### Failure pattern (Figure 7)

**Figure 7:**
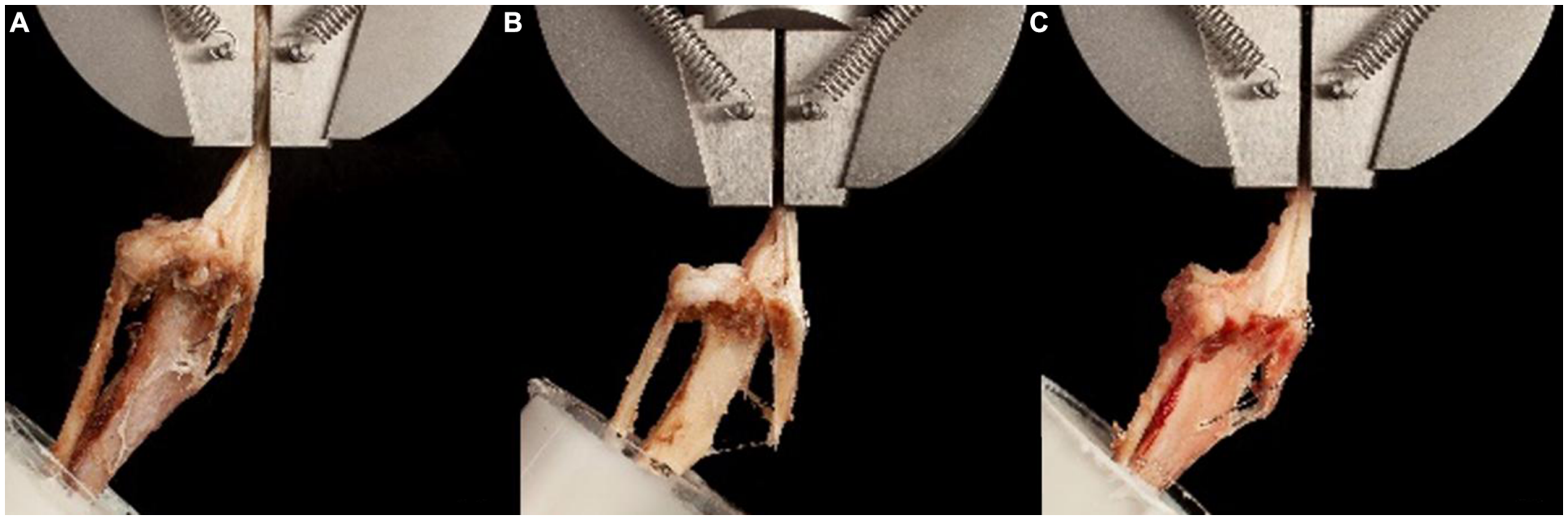
Photographs of the most common failure pattern for each treatment group. **(A)** Group 1 (tension modified-hemicerclage): Slight displacement and spreading of the osteotomy fragment. Complete failure at the breakage point of the cerclage. **(B)** Group 2 (2Pins): Spreading of the osteotomy distally after fracture of the periosteal bridge, resulting in extraction of the pins. **(C)** Group 3 (2Pins + TBW): Minimal displacement of the osteotomy fragment, until the breakage of the cerclage. Complete failure and extraction of the pins occurred soon after, similar to Group 2.

The most common failure pattern for MH constructs was severing the osteotomy fragment vertically by the wire, followed by wire breakage and patellar ligament rupture. After failure of the periosteal bridge, the osteotomy fragment displaced proximally in 3/10 prior to complete failure. In 7/10 specimens, complete failure occurred simultaneously or immediately after breaking of the periosteal bridge. For 2Pins constructs, four constructs failed due to pins severing the osteotomy fragment while remaining in the tibia, five due to pin pullout, and one due to a combination of one pin pulling out and the other severing the osteotomy fragment. The video analysis revealed that the constructs became unstable as soon as the periosteal bridge broke, and the tuberosity segment began separating from the main tibial segment at the distal aspect of the osteotomy immediately after the breakage of the periosteal bridge. Among the 2Pins + TBW group, the most common source of complete failure was simultaneous TBW breakage and pin pullout (*n* = 4) or TBW breakage followed by secondary pin pullout (*n* = 3). One specimen failed by fracturing the tibia in an oblique fashion at the anchor point of the TBW. One specimen failed after TBW breakage and simultaneous proximal tibial fracture. One specimen fractured at the level of the pot while the implants remained intact. In the 2Pins + TBW group, the video analysis showed that the osteotomy fragment was only slightly displaced prior to complete failure in most specimens. Fragment displacement was primarily vertical, with 4 specimens showing only slight spreading of the osteotomy.

The main modes of failure are summarized in Table 1.

## Discussion

In this study we found that a novel TTT fixation technique with a single orthopedic wire placed as a tension modified hemicerclage was not different from a 2Pins + TBW technique in stiffness and load at failure. Both of these techniques were stronger than the 2Pins technique. The forces recorded at complete failure in the 3 treatment groups were within the normal range of the quadriceps muscle forces, which was estimated to be approximately 94.8% of a patient’s body weight during walking (31). This result suggests that all three techniques may be strong enough to withstand normal forces on the patellar ligament during walking. However, mechanical failure data collected in a cadaveric study are difficult to interpret clinically. Further studies are warranted to determine the safety and efficacy of the novel technique in clinical cases.

The load to failure reported in our study was approximately two times higher than that reported in a similar study performed with tibiae from raccoon dogs, which have similar mean body weights to the specimens used in the present study (12). The raccoon dog study reported mean weights of 7.1 kg and 6.2 kg. In comparison, our study presented a mean weight of 7.4 kg for the specimens. The mean stiffness, similarly, was higher in the constructs in the current study. A notable difference was observed in the mode of failure: our study had fewer instances of tibial fractures compared to the raccoon dog study. The occurrence of bone fractures at lower forces in the raccoon dog study may be attributed to several factors. These include inherent species-based differences in bone quality, variations in the age of the specimens, and the methodologies used for specimen storage and preparation, particularly freeze/thaw processes (29, 32, 33). Such factors can significantly impact the biomechanical properties of the bones, thereby influencing the outcomes of biomechanical testing.

Our results confirmed that 2 pins combined with a tension band counteract the tensile force applied by the patellar ligament and therefore withstands greater forces than 2 pins alone. Similarly, based on our results, we theorize that the MH counteract tensile forces similarly to TBW, because the wire loops both in a craniocaudal and mediolateral direction, mimicking the direction of a pin and a TBW (Figure 2). Thus, this construct offers similar biomechanical advantages to TBW without the need for pins that may migrate or cause morbidity (14, 15, 20).

While the implant size would be modified on a case-by-case basis in clinical cases, (7, 34) we elected to use standardized implant sizes, including 1.0 mm 316LVM pins and/or 0.6 mm stainless steel orthopedic wire, across all specimens. While 1.0 mm diameter pins are small, the presence of fragment fractures as a mode of failure in the current study may indicate that two 1.0 mm pins horizontally aligned might have been oversized for some of the specimens. Alternatively, smaller pins or vertical alignment of the pins may be preferable in small breed dogs in hopes of lessening the incidence of tibial tuberosity fracture if a construct utilizing pins through the tuberosity is going to be used (18).

The observed modes of failure in our study were notably distinct for each fixation group, shedding light on the biomechanical behaviors of different techniques. The single orthopedic wire group exhibited failure primarily at the site where the wire intersects the tuberosity, highlighting potential concerns about the structural integrity of the tuberosity under load. In contrast, the 2Pin group’s primary mode of failure was pin pullout, suggesting that this method may not provide sufficient anchorage. The 2Pin + TBW group showed failure through both wire breakage and pin pullout, indicating a combination of material and anchorage weaknesses. This variance in failure modes is intriguing when compared to other studies. For instance, in a study evaluating pins and TBW, the primary failure mode involved the untwisting of the wire and bending of the pins, rather than breakage or pullout (28). Similarly, another study focusing on 2Pins and 2Pins + TBW constructs reported patellar ligament failure as the main issue (18) which was not observed in our study at all. Possible reasons for differences in the mode of failure are the smaller specimen size, lower stiffness, inherent variability in conformation, potential differences in specimen handling such as freeze–thaw cycles, differences in surgical technique such as pin orientation or wire twisting, and differences in bone quality between specimens. These potential factors underline the complexity of biomechanical testing and the challenge of directly comparing different studies. It is crucial to consider these variables when interpreting our results and those of similar research.

The results of this *ex vivo* cadaveric study should be interpreted considering some limitations. The unidirectional tensile load-to-failure mechanical testing does not replicate physiologic loading or cyclic fatigue encountered in the postoperative convalescent period. Therefore, translation of the results to a clinical setting should be done with caution. Another limitation is the broad range of body weight of the dogs used for the study. We selected small breed dogs because of the clinical significance, which may be considered more relevant in comparison to other similar studies that utilized larger cadavers (12, 18, 28–30). The wide range of tibia size would likely have caused variability in mean stiffness and maximum load at failure, as well as failure modes. Our results show that the proposed technique can withstand tensile loads similar to a tension band wire construct. However, there are uncertainties regarding the behavior of the wire within the crest fragment and its interaction with trabecular bone density, potentially affecting the final position of the fragment. Future studies, such as CT imaging, will be necessary to measure the degree of lateral displacement. Nonetheless, our study focuses on defining the biomechanical properties of the construct. Despite these limitations, the results from the present study are comparable to previously reported studies, when differences in weight categories are taken into account (12, 18, 28–30). A direct comparison to other studies should be made with caution due to the differences in specimen weight range, the small sample size, breed/species, and, consequently, implant size.

In framing the results of our study, it’s essential to emphasize that this investigation represents an initial biomechanical assessment, primarily focused on establishing the basic mechanical viability of the novel TTT fixation technique using a single orthopedic wire. This approach aligns with the foundational step in introducing any new surgical method, where the primary concern is to establish that the technique is mechanically sound and can at least withstand normal physiological loads. By demonstrating comparable stiffness and load at failure with the 2Pins + TBW method, and superior performance compared to the 2Pins method, our study suggests that the novel technique is mechanically stable enough for further consideration. For the sake of consistency and comparability with existing biomechanical literature, we conducted our tests with the stifle positioned at 135° extension, as established in previous studies (12, 18, 28, 30). A consideration when applying the modified hemicerclage technique is the potential for soft tissue trauma, primarily because the tibialis cranialis muscle must be carefully prepared to facilitate the passage and retrieval of the wire. However, based on our clinical experience, the extent of trauma incurred is generally acceptable and can be effectively managed within the surgical process.

This study reported a novel fixation technique for TTT that utilizes a single-length orthopedic wire placed as a modified hemicerclage in a tension band fashion. The stiffness and load at failure of the constructs stabilized using this new technique were not different from the constructs stabilized with 2Pins + TBW. These results suggest that this new technique may have potential benefits, such as reducing implant-associated complications. It is important to underline that while our study contributes valuable initial data regarding the mechanical stability of the new fixation method, it cannot be considered conclusive regarding the technique’s clinical applicability. The results point toward a need for further research, particularly involving clinical trials. Such studies would not only provide more comprehensive data on the method’s mechanical resilience over time but also offer insights into potential advantages regarding reduced necessity for implant removal, a key consideration given the complications associated with traditional pin-based methods.

## Data availability statement

The raw data supporting the conclusions of this article will be made available by the authors, without undue reservation.

## Ethics statement

Ethical approval was not required for the studies involving animals in accordance with the local legislation and institutional requirements because Tibias from skeletally mature dogs, euthanatized for reasons unrelated to this study, were collected for the study. All the cadaveric specimens were donated for research purposes by their owners, and written consent was obtained according to the institute and national regulations. Written informed consent was obtained from the owners for the participation of their animals in this study.

## Author contributions

PN: Writing – review & editing, Writing – original draft, Visualization, Validation, Project administration, Methodology, Investigation, Formal analysis, Data curation, Conceptualization. RC: Writing – review & editing, Writing – original draft, Visualization, Project administration, Investigation, Formal analysis, Data curation. NK: Methodology, Writing – review & editing, Writing – original draft, Visualization, Validation, Conceptualization. AP: Writing – review & editing, Writing – original draft, Visualization, Validation, Supervision, Resources, Methodology. ST: Validation, Supervision, Methodology, Writing – review & editing, Writing – original draft, Visualization. BP: Writing – review & editing, Writing – original draft, Visualization, Validation, Supervision, Software, Resources, Project administration, Methodology, Investigation, Formal analysis, Data curation, Conceptualization.

## Footnotes

^1^IBM Corp. Released 2020. IBM SPSS Statistics for Windows, Version 27.0. Armonk, NY: IBM Corp.
